# Multi-View Spectral Clustering Based on Multi-Smooth Representation Fusion for Cancer Subtype Prediction

**DOI:** 10.3389/fgene.2021.718915

**Published:** 2021-09-06

**Authors:** Jian Liu, Shuguang Ge, Yuhu Cheng, Xuesong Wang

**Affiliations:** ^1^School of Information and Control Engineering, China University of Mining and Technology, Xuzhou, China; ^2^Engineering Research Center of Intelligent Control for Underground Space, Ministry of Education, China University of Mining and Technology, Xuzhou, China

**Keywords:** multi-view clustering, cancer subtypes prediction, multi-omics data, spectral clustering, smooth representation, graph fusion

## Abstract

It is a vital task to design an integrated machine learning model to discover cancer subtypes and understand the heterogeneity of cancer based on multiple omics data. In recent years, some multi-view clustering algorithms have been proposed and applied to the prediction of cancer subtypes. Among them, the multi-view clustering methods based on graph learning are widely concerned. These multi-view approaches usually have one or more of the following problems. Many multi-view algorithms use the original omics data matrix to construct the similarity matrix and ignore the learning of the similarity matrix. They separate the data clustering process from the graph learning process, resulting in a highly dependent clustering performance on the predefined graph. In the process of graph fusion, these methods simply take the average value of the affinity graph of multiple views to represent the result of the fusion graph, and the rich heterogeneous information is not fully utilized. To solve the above problems, in this paper, a Multi-view Spectral Clustering Based on Multi-smooth Representation Fusion (MRF-MSC) method was proposed. Firstly, MRF-MSC constructs a smooth representation for each data type, which can be viewed as a sample (patient) similarity matrix. The smooth representation can explicitly enhance the grouping effect. Secondly, MRF-MSC integrates the smooth representation of multiple omics data to form a similarity matrix containing all biological data information through graph fusion. In addition, MRF-MSC adaptively gives weight factors to the smooth regularization representation of each omics data by using the self-weighting method. Finally, MRF-MSC imposes constrained Laplacian rank on the fusion similarity matrix to get a better cluster structure. The above problems can be transformed into spectral clustering for solving, and the clustering results can be obtained. MRF-MSC unifies the above process of graph construction, graph fusion and spectral clustering under one framework, which can learn better data representation and high-quality graphs, so as to achieve better clustering effect. In the experiment, MRF-MSC obtained good experimental results on the TCGA cancer data sets.

## Introduction

Cancer is a malignant and heterogeneous disease caused by changes in cellular and molecular expression, epigenetics, transcription, and proteome levels (Burrell et al., 2013). This heterogeneity is reflected in the fact that the same type of cancer will produce subtypes with different representations, which will further affect the clinical treatment plan and prognosis (Bedard et al., 2013). With the development and maturity of the new generation of sequencing technologies, a large number of multi-omics biological data have been collected in some public data sets and are easily accessible to researchers (Schuster, 2008). The Cancer Genome Atlas (TCGA) is a landmark cancer genomics project that stores biological information including mRNA expression data, methylation data, miRNA expression data, and gene mutation data from more than 30 type of cancers and thousands of cancer patients. Therefore, it is particularly important to build a clustering model that makes full use of these biological information to solve the problem of discovering cancer subtypes (Akbani et al., 2014).

In recent years, some effective multi-view clustering methods have been designed and applied to biological data (Shen et al., 2010; Zhang et al., 2012; Mo et al., 2013; Wang et al., 2014; Meng et al., 2016; Ma and Zhang, 2017; Shi et al., 2017; Guo et al., 2019; Yu et al., 2019). In order to achieve the task of clustering, scholars initially focused on feature selecting and feature dimensionality reduction techniques. They all used different strategies to transform or project high-dimensional data into low-dimensional feature space and then realized clustering through K-means. For example, iCluster (Shen et al., 2010) is a Gaussian hidden variable model, and its extended version, iClusterPluse (Mo et al., 2013), is an effective and classical multi-omics data clustering method. It considers that different variable types follow different linear probability relationships, and then constructs a joint sparse model to complete feature selecting and sample clustering tasks. However, iClusterPlus has an obvious drawback: it includes a pre-selecting process for genes that filters out important information, and the clustering results are sensitive to this operation. In order to solve the problem of data preprocessing, many classical dimensionality reduction techniques are applied to the proposed clustering algorithms, e.g., Principal Component Analysis (PCA; Ding and He, 2004), Non-negative Matrix Factorization (NMF; Zhang et al., 2012), etc. Shi et al. (2017) applied the improved PCA to design Pattern Fusion Analysis (PFA) method, which projects each data set into a low-dimensional feature space with local patterns while reducing noise. Then PFA uses the dynamic collimation algorithm to achieve the fusion of feature space.

The above methods only focus on the characteristics of each kind of omics data, without considering the structural characteristics of the data, which can reveal the potential similarity between samples and has great guiding significance for the study of data representation. Considering that the sample (patient) size of the biological data is much smaller than the feature (gene) size, some methods for cancer subtype prediction based on graph learning have been designed. Based on cancer samples, graph learning can quickly construct similar graphs and eventually transform them into spectral clustering problems to achieve clustering. For example, Wang et al. (2014) proposed a widely used clustering algorithm for multi-omics data, named as Similarity Network Fusion (SNF). SNF uses the exponential similarity kernel method to construct a sample similarity network for each data type instead of the dimensionality reduction process, and then uses the nonlinear information fusion technology to integrate these networks into a single similarity network. Inspired by SNF, Ma and Zhang (2017) proposed Affinity Network Fusion (ANF) method, which constructs K-nearest neighbor similar networks of patients for each data type, and then fused these networks based on random step size method. Other algorithms based on graph learning are also very effective in the recognition of cancer subtypes. For example, Yu et al. (2019) proposed Multi-view Clustering using Manifold Optimization (MVCMO), which uses linear search on Stiefel manifold space to solve the spectral clustering optimization problem.

The above methods all use the original omics data matrix to construct the similarity matrix, and fuse the obtained multiple similarity matrices, ignoring the learning of the similarity matrix. In the process of graph fusion, the similarity between sample points is usually different in different views. Some existing algorithms simply take the average value of the affinity graph of multi-omics to represent the result of the fusion graph, and the rich heterogeneous information is not fully utilized. In addition, most of the graph-based multi-view clustering methods separate the data clustering process from the graph learning process, which makes the graph construction independent of the clustering task, leading to the clustering performance highly dependent on the predefined graph. In this paper, we design a Multi-view Spectral Clustering method based on Multi-smooth Representation (MRF-MSC) for the exploration of cancer subtypes. MRF-MSC combines graph learning, graph fusion and spectral clustering into one framework to avoid the above problems. Firstly, MRF-MSC uses the graph regularization method to calculate the smooth representation of each omics data type. The original feature space raw data can be effectively projected into the corresponding sample similarity subspace. The smooth representation can explicitly enhance the grouping effect, that is, it enhances the similarity between samples of the same category and reduces the similarity between samples of different categories (Hu et al., 2014). Secondly, the multi-smooth representation matrices of multi-omics data are integrated to form a fused similarity matrix. Considering that each omics data is of different importance to the prediction of cancer subtypes, MRF-MSC adaptively weights the smooth regularization representation of each omics data by using the self-weighting method in the process of graph fusion. Finally, MRF-MSC optimizes the fused similarity matrix through constrained Laplacian rank to learn a new block diagonal matrix with *k* connected components (*k* is the number of classes), which is beneficial for clustering. This problem can be solved by using spectral clustering (Ng et al., 2001). Spectral clustering is a classical data clustering method and widely used in multi-view clustering algorithms (Nie et al., 2016; Kang et al., 2020; Feng et al., 2021; Ge et al., 2021) recently. In order to verify the effectiveness of MRF-MSC, cancer subtypes prediction experiments were carried out on TCGA data sets. The results showed that MRF-MSC was able to obtain more significant clinical differences in cancer typing. In the Breast Invasive Carcinoma (BRCA) analysis, the MRF-MSC results validated previous clinical studies and identified biologically significant cancer subtypes.

## Materials and Methods

**FIGURE 1:**
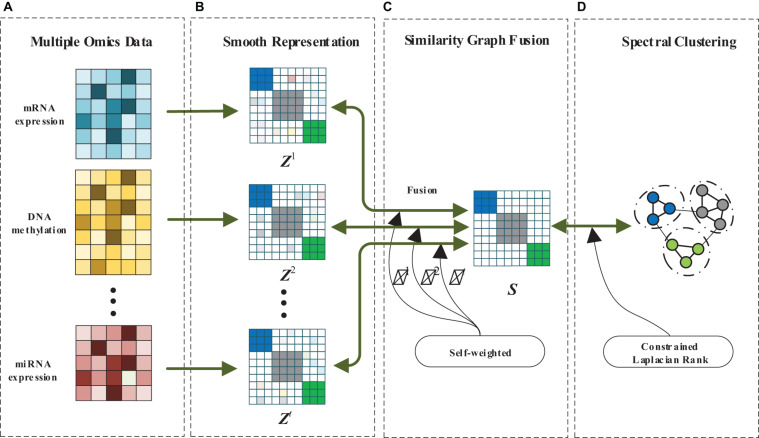
The framework of MRF-MSC. **(A)** Multiple omics data. **(B)** Smooth representation. **(C)** Similarity graph fusion. **(D)** Spectral clustering.

In this paper, we design a MRF-MSC for cancer subtypes prediction. The framework of MRF-MSC as shown in Figure 1. Given multi-omics data sets, we first calculate the similarity matrix with smooth representation for each data set to measure the similarity between sample points. Then, the graph fusion and self-weighted methods are used to integrate the multi-smooth representation into a fused similarity matrix. Finally, constrained Laplacian rank and spectral clustering are adopted to optimize the fused similarity matrix, and the clustering results can be obtained.

### Smooth Representation of Multi-Omics Data

Given a set of cancer multi-omics data ***X*** = {***X***^1^, ***X***^2^, ⋯, ***X***^*t*^}, ***X***^*v*^ ∈ ℝ^*m*^*v*^×*n*^, where *t* is the number of data sets, ***X***^*v*^ is the *v*-th omics data, *m*^*v*^ indicates that the *v*-th dataset has *m* features, *n* is the number of samples. In order to obtain the final fused similarity graph, we need to calculate the similarity matrix of each omics data ***Z*** = {***Z***^1^, ***Z***^2^, ⋯, ***Z***^*t*^}, ***Z***^*v*^ ∈ ℝ^*n*×*n*^. This enables the raw omics data to be aggregated into their respective subspaces.

Take a single omics data ***X***^*v*^ as an example, we introduce a self-representation method to measure the similarity between samples:

(1) $${\text{X}}^{v}={\text{X}}^{v}{\text{Z}}^{v}+{\text{E}}^{v}$$

where ***Z***^*v*^ is coefficient matrix which encodes the similarity between the data samples, *E*^*v*^ is error matrix. For Eq. 1, we explicitly strengthen the grouping effect between samples by smooth representation. This can enhance the similarity between samples of the same category and reduce the similarity between samples of different categories. The smooth representation can be roughly written as

(2) $$\min_{Z^{v}}{||{\text{X}}^{v}-{\text{X}}^{v}{\text{Z}}^{v}||}_{F}^{2}+\alpha \Omega \left({\text{Z}}^{v}\right)s.t.{\text{Z}}^{v}\geq 0$$

where α is a hyperparameter, Ω is the regularization term of the smooth representation. If two sample points are close to each other in the original feature space, then they should also maintain this property in the new feature space. That is, for samples *i* and *j*, the following rules should be satisfied: ${||{\text{x}}_{i}^{v}-{\text{x}}_{j}^{v}||}_{2}\to 0\Rightarrow {||{\text{z}}_{i}^{v}-{\text{z}}_{j}^{v}||}_{2}\to 0$, where ***x^v^*** and ***z^v^*** is the vector of *X*^*v*^ and *Z*^*v*^, respectively. The smooth representation regularization term in Eq. 2 can be defined as

(3) $$\Omega \left({\text{Z}}^{v}\right)=\frac{1}{2}\sum_{i=1}^{n}\sum_{j=1}^{n}{\text{w}}_{ij}^{v}{||{\text{z}}_{i}^{v}-{\text{z}}_{j}^{v}||}_{2}=\text{tr}\left({\text{Z}}^{v}{\text{L}}^{t}{\left({\text{Z}}^{v}\right)}^{T}\right)$$

where tr is the trace of the matrix, and T is the transpose of the matrix, ${\text{w}}_{ij}^{v}$ is an element in the weight matrix ***W***^*v*^ that measures the similarity between sample points. ***L***^*v*^ = ***D***^*v*^−***W***^*v*^ is the Laplacian matrix, where ***D***^*v*^ is a diagonal degree matrix which diagonal elements satisfy ${\text{d}}_{ii}^{v}=\sum_{j=1}^{n}{\text{w}}_{ij}^{v}$. Now, there’s a lot of ways to calculate *W*^*v*^. Here, we construct *W*^*v*^ by using the most common used K-nearest neighbor method. Finally, Eq. 2 can be written as:

(4) $$\min_{Z^{v}}{||{\text{X}}^{v}-{\text{X}}^{v}{\text{Z}}^{v}||}_{F}^{2}+\alpha \text{tr}\left({\text{Z}}^{v}{\text{L}}^{v}{\left({\text{Z}}^{v}\right)}^{\text{T}}\right)s.t.Z^{v}\geq 0$$

Through Eq. 4, the smooth representation ***Z***^*v*^ of each omics data can be obtained.

### The Fusion of Multi-Smooth Representations

How to integrate similar graphs in graph learning and make full use of the information of different data sets is the key of multi-view clustering method. After obtaining smooth representations *Z* = {***Z***^1^, ***Z***^2^, ⋯, ***Z***^*t*^} of multi-omics data, we want to learn a fused similarity graph ***S*** that minimizes the difference between ***S*** and ***Z***^*v*^. Then the graph fusion process of multi-smooth representations can be denoted as:

(5) $$\min_{Z^{v},S}\sum_{v=1}^{t}{||\text{S}-{\text{Z}}^{v}||}_{F}s.t.{\text{Z}}^{v}\geq 0$$

Considering that each omics data is of different importance to the prediction of cancer subtypes, we assign weighting factors ε = {ε^1^, ε^2^, ⋯, ε^*t*^} to ***Z*** = {***Z***^1^, ***Z***^2^, ⋯, ***Z***^*t*^}. ε^*v*^ describes the contribution of the *v*-th smooth representation of each omics data to the graph fusion task. If ***Z***^*v*^ is closer to ***S***, then its corresponding contribution weight ε^*v*^ is larger, which can reduce the impact of poor quality smooth representation on ***S***. Here, we adopt the self-weighting method in Nie et al. (2017) to carry out adaptive weighting for the smooth representation. The weighting factor of each smooth representation can be automatically tuned without any additional parameters.

Take the derivative of ***Z***^*v*^ in Eq. 5 and set the derivative to zero, we have

(6) $$\sum_{v=1}^{t}\varepsilon^{v}\frac{\partial \left({||\text{S}-{\text{Z}}^{v}||}_{F}\right)}{\partial {\text{Z}}^{v}}=0$$

where

(7) $$\varepsilon^{v}=\frac{1}{2\left({||\text{S}-{\text{Z}}^{v}||}_{F}\right)}$$

Since ε^*v*^ is calculated by ***Z***^*v*^, Eq. 6 cannot be solved directly. However, if ε^*v*^ is assigned a fixed value as the weighting factor of each smooth representation, then Eq. 6 can be used to solve the following problems:

(8) $$\min_{Z^{v},S}\sum_{v=1}^{t}\varepsilon^{v}{||\text{S}-{\text{Z}}^{v}||}_{F}^{2}s.t.{\text{Z}}^{v}\geq 0$$

In Eq. 8, since both ***Z***^*v*^ and ***S*** are goals to be solved, we cannot directly optimize the objective function. We can obtain the objective function of multi-smooth representation fusion by combining Eqs 4, 5 as:

(9) $$\min_{Z^{v},S}\sum_{v=1}^{t}\left({||{\text{X}}^{v}-{\text{X}}^{v}{\text{Z}}^{v}||}_{F}^{2}+\alpha \text{tr}\left({\text{Z}}^{v}{\text{L}}^{v}{\left({\text{Z}}^{v}\right)}^{\text{T}}\right)+\beta \varepsilon^{v}{||\text{S}-{\text{Z}}^{v}||}_{F}^{2}\right)s.t.{\text{Z}}^{v}\geq 0$$

where β is a hyperparameter.

By solving the above problem, we can learn the smooth representations and fused similarity graph of multi-omics data. In addition, the smooth representation is dynamically weighted during the fusion process, which effectively reduces the influence of the smooth representation of low-quality omics data on the fused similarity graph.

### Multi-View Spectral Clustering Based on Multi-Smooth Representation Fusion

After calculating the fused similarity graph ***S***, although we can directly cluster ***S*** based on spectral clustering, the ***S*** obtained by Eq. 9 may not be optimal for the final clustering task. So, we attempt to optimize the clustering structure of *S*.

Ideally, a graph that is best for clustering tasks should have exactly *k* connected components, that is, data points are formed into *k* clusters. This can be done according to the following theorem.

**Theorem 1**. The number of connected components *k* of the graph ***S*** is equal to the multiplicity of zero eigenvalues of its Laplacian matrix *L*_*S*_.

Since the elements in ***S*** are non-negative, then ***L_S_*** is a positive semi-definite matrix. Denote σ_*i*_(***L_S_***) is the *i*-th minimum eigenvalue of ***L_S_***, we can obtain the optimal solution of *S* through the following constrained Laplacian rank method: $\sum_{i=1}^{k}\sigma_{i}\left({\text{L}}_{S}\right)=0$ and rank(***L***_*S*_) = *n* − *k*, where rank(*L*_*S*_) is the rank of *L*_*S*_. By Ky Fan’s theorem (Fan, 1949), we have

(10) $$\sum_{i=1}^{k}\sigma_{i}\left({\text{L}}_{S}\right)=\min_{F,F^{\text{T}}F=I}tr\left({\text{F}}^{\text{T}}{\text{L}}_{S}\text{F}\right)$$

where ***F*** is the first *k* minimum eigenvalues correspond to eigenvectors of ***L_S_***. The right side of Eq. 10 is the objective function of spectral clustering. Therefore, Eq. 10 establishes the connection between the desired fused graph structure and spectral clustering. The optimization of Eq. 10 results in the fused similarity graph ***S*** with exact *k* connected components.

According to Eqs 9, 10, we combine the smooth representation of multi-omics data, the fusion of multi-smooth representation and multi-view spectral clustering into one framework, and propose the MRF-MSC. The objective function of MRF-MSC can be written as

(11) $$\begin{array}{c} \min_{Z^{v},S,F}\sum_{v=1}^{t}(||{\text{X}}^{v}-{\text{X}}^{v}{\text{Z}}^{v}|{|}_{F}^{2}+\alpha \text{tr}\left({\text{Z}}^{v}{\text{L}}^{v}{\left({\text{Z}}^{v}\right)}^{\text{T}}\right)+\\ \beta \varepsilon^{v}||\text{S}-{\text{Z}}^{v}|{|}_{F}^{2})+\lambda tr({\text{F}}^{\text{T}}{\text{L}}_{S}\text{F})s.t.\text{Z}{}^{v}\geq 0,\text{F}{}^{T}\text{F}=I \end{array}$$

where α, β, and λ are hyperparameters.

We conclude that MRF-MSC has the following advantages in predicting cancer subtypes using multi-omics data.

(1) The characteristic of biological data is that the sample size is much smaller than the feature size. The smooth representation of the omics data not only retains the characteristic of the original data, but also effectively obtains the similarity between the sample points, which provides a relatively high quality subspace representation for the subsequent graph fusion process.
(2) In general, multi-omics data come from different platforms, which leads to different contribution of each omics data to clustering results. In the process of similar graph fusion, MRF-MSC uses self-weighting to perform multi-smooth representation fusion. In this way, the complementarity of various biological information is realized, the influence of noise data is reduced, and the quality of fused similar graph is improved.
(3) We introduce spectral clustering into MRF-MSC, which can improve the accuracy of the final result. In this joint MRF-MSC framework, the constrained Laplacian rank is used to constrain the structure of the fusion similar graph to obtain a graph structure that is conducive to the clustering task. Moreover, we use the learned graph structure to guide the construction of the graph, so that this mutual learning and iterative method can improve the final clustering result.

### Optimization of MRF-MSC

We can optimize ***Z***^*v*^, ***S*** and ***F*** step by step according to Eq. 11 through the idea of iterative optimization.

(1) Fixing ***S*** and ***F*** to solve ***Z***^*v*^

Based on Eq. 11, we can get the objective function Eq. 9 about ***Z***^*v*^. It is observed that in Eq. 9, ***Z***^*v*^ is independent for each omics data. Therefore, we can update ***Z***^*v*^ separately for each omics data. Taking the derivative of ***Z***^*v*^ in Eq. 9, we have

(12) $$\left({\left({\text{X}}^{v}\right)}^{\text{T}}{\text{X}}^{v}+\beta \varepsilon^{v}\text{I}\right){\text{Z}}^{v}+\alpha {\text{Z}}^{v}{\text{L}}^{v}={\left({\text{X}}^{v}\right)}^{\text{T}}{\text{X}}^{v}+\beta \varepsilon^{v}\text{S}$$

The above equation is a standard Sylvester equation with unique solution. We can easily get the solution result of ***Z***^*v*^:

(13) $${\text{Z}}^{v}={\left({\left({\text{X}}^{v}\right)}^{\text{T}}{\text{X}}^{v}+\beta \varepsilon^{v}\text{I}+\alpha {\text{L}}^{v}\right)}^{-1}\left({\left({\text{X}}^{v}\right)}^{\text{T}}{\text{X}}^{v}+\beta \varepsilon^{v}\text{S}\right)$$

(2) Fixing ***Z***^*v*^ and ***F*** to solve ***S***

Based on Eq. 11, we can get the objective function of ***S*** as follows:

(14) $$\min_{S}\sum_{v=1}^{t}\beta \varepsilon^{v}{||\text{S}-{\text{Z}}^{v}||}_{F}^{2}+\lambda tr\left({\text{F}}^{\text{T}}{\text{L}}_{S}F\right)$$

According to $tr\left({\text{F}}^{\text{T}}{\text{L}}_{S}\text{F}\right)=\sum_{i,j}\frac{1}{2}{||f_{i}-f_{j}||}_{2}^{2}s_{ij}$, where *s*_*ij*_ is the elements of *S*, we define $g_{ij}={||f_{i}-f_{j}||}_{2}^{2}$ and *g*_*i*_ is a vector whose *j*-th element equal to *g*_*ij*_. So, the Eq. 14 can be calculated by column

(15) $$\min_{s_{i}}\sum_{v=1}^{t}\beta \varepsilon^{v}{||{\text{s}}_{i}-{\text{z}}_{i}^{v}||}_{F}^{2}+\frac{\lambda }{2}{\text{g}}_{i}^{\text{T}}{\text{s}}_{i}$$

Taking the derivative of *s*_*i*_ in Eq. 15, we can obtain the solution of *s*_*i*_:

(16) $${\text{s}}_{i}=\frac{\sum_{v=1}^{t}\varepsilon^{v}{\text{z}}_{i}^{v}-\frac{\lambda g_{i}}{4\beta }}{\sum_{v=1}^{t}\varepsilon^{v}}$$

(3) Fixing ***Z***^*v*^ and ***S*** to solve ***F***

Based on Eq. 11, we can get the objective function of *F* as follows:

(17) $$\min_{F}\lambda tr\left({\text{F}}^{\text{T}}{\text{L}}_{S}\text{F}\right)s.t.{\text{F}}^{\text{T}}\text{F}=\text{I}$$

In the above formula, the optimal solution of *F* is the *k* eigenvectors corresponding to the first *k* minimum eigenvalues. After the iterative optimization, we take each row of the final *F* as a new representation of each sample, and use the K-means algorithm to calculate the clustering results.

We use pseudo-code to summarize the MRF-MSC solution process in Algorithm 1.

**Table:** 

**Algorithm 1**: MRF-MSC algorithm.
**Input:** cancer multi-omics data ***X*** = {***X***^1^, ***X***^2^, ⋯, ***X***^*t*^}, the number of cancer subtypes *k*, the maximum number of iterations MaxIter, *K* is the number of neighbors in KNN, hyperparameters α, β and λ. **Output:** smooth representation of each omics data ***Z***^*v*^, fused similarity graph ***S***, eigenvectors ***F***.
Initialize ***S*** = **I**, ε^*v*^ = 1/*t*. **Repeat** Update ***Z***^*v*^ according to Eq. 13, Set $z_{ij}^{v}=\max\left(z_{ij}^{v},0\right)$ for every element $z_{ij}^{v}$ in ***Z***^*v*^, Update ***S*** according to Eq. 16, Update ***F*** by optimizing Eq. 17 Update ε^*v*^ according to Eq. 7, **Until** meeting stop condition Stop condition: the maximum number of iterations MaxIter is reached or the relative change of ***S*** is less than 10^–3^.

## Results and Discussion

### Multi-Omics Data Sets

In order to prove the effectiveness of the MRF-MSC algorithm in cancer subtype prediction, we applied MRF-MSC to the cancer multi-omics data downloaded and preprocessed from TCGA by Wang et al. (2014) and Rappoport and Shamir (2018). We conducted experiments on five cancer types: BRCA, Glioblastoma Multiforme (GBM), Lung Squamous Cell Carcinoma (LSCC), Kidney Renal Clear Cell Carcinoma (KIRC), and Colon Adenocarcinoma (COAD). Each cancer contains three types of cancer expression data from different platforms: mRNA expression, DNA methylation, and miRNA expression. The details on five types of cancer multi-omics data sets in Wang et al. (2014) and Rappoport and Shamir (2018) are shown in Tables 1, 2, respectively. For these cancer types, we also downloaded the patient’s clinical information, including all cancer survival data, and BRCA somatic mutation data, copy number data, and clinical data of drug treatments for subsequent analysis and algorithm comparison. The clinical information of BRCA was downloaded from the cBioPortal database.^1^

**TABLE 1:** Detailed information on five types of cancer multi-omics data sets in Wang et al. (2014).

Cancer type	Number of genes			Number of samples
	mRNA	Methylation	miRNA	
GBM	12,042	1,305	534	215
BRCA	17,814	23,094	354	105
KIRC	17,899	24,960	329	122
LSCC	12,042	23,074	352	106
COAD	17,814	23,088	312	92

**TABLE 2:** Detailed information on five types of cancer multi-omics data sets in Rappoport and Shamir (2018).

Cancer type	Number of genes			Number of samples
	mRNA	Methylation	miRNA	
GBM	12,042	5,000	534	271
BRCA	20,531	5,000	1,046	622
KIRC	20,531	5,000	1,046	181
LSCC	20,531	5,000	1,046	337
COAD	20,531	5,000	705	213

### Evaluation Metrics

We chose the *P*-value based on the Cox log-rank model in the survival analysis of cancer subtype prediction to measure the MRF-MSC algorithm. For the characteristic that cancer samples have no real labels, it is impossible to use accuracy to evaluate the clustering results. In this case, survival analysis is necessary to verify the degree of difference between cancer subtypes (Mantel, 1966). We established a Cox regression model to obtain the *P*-value of the log-rank test of survival separation (Goel et al., 2010). If the *P*-value is smaller, it means that the survival rate between different clusters is more significant. Furthermore, it shows that the greater the difference between clusters, the more likely it is to get potential cancer subtypes with different characteristics.

### Comparison Algorithms and Parameter Settings

For comparison, we selected five effective multi-view clustering algorithms in the field of cancer subtype prediction as the comparison algorithm: iClusterPlus, PFA, SNF, ANF, and MVSCO. Their details are as follows.

(1) iClusterPlus (Mo et al., 2013). iClusterPlus considers that different variable types follow different linear probability relationships, and then constructs a joint sparse model to complete the task of sample clustering and feature selection.
(2) PFA (Shi et al., 2017). PFA first uses the method of local information extraction to project each omics data in a low-dimensional space. Then, based on the idea of manifold learning, a dynamic collimation method is constructed to integrate low-dimensional spatial information into the fused feature space. Finally, the K-means method is used to find the label of the sample.
(3) SNF (Wang et al., 2014). SNF first uses the exponential similarity kernel method to define the similarity between the sample points of each omics data. Then, it uses the K-nearest neighbor method and a complete sparse kernel measurement method to obtain the local similarity graph and the global similarity graph of each omics data, respectively. Finally, the information transfer model based on the random walk idea is used to fuse the local information and the global information. Furthermore, spectral clustering method is used to cluster the fused graph.
(4) ANF (Ma and Zhang, 2017). PFA is an improved version of SNF. It constructs a K-nearest neighbor similar network for each omics data, and then merges these networks based on the random step method.
(5) MVSCO (Yu et al., 2019). MVSCO first draws on the method of Zhang et al. (2012) to find the similarity between sample points of each omics data, and then uses the current search method in the Stiefel manifold space to optimize the multi-view spectral clustering problem. Finally, the K-means method is used to predict the label of the sample.

Here, we present the parameter selection range of MRF-MSC algorithm and all comparison algorithms. Three hyperparameter α, β and λ in MRF-MSC are set to α, β, λ ∈ [10^−6^, 10^6^]. iClusterPlus has two penalty parameters α and λ, where α is set to 1 and λ is obtained by automatic learning. In MRF-MSC, SNF, ANF, and MVSCO methods, the number of neighbors of KNN is set to *K* ∈ [5,50]. The hyperparameter α in SNF is set to α ∈ [0.3,0.8]. We used the default parameter to run PFA algorithm.

### Results on Cancer Multi-Omics Data Sets

**FIGURE 2:**
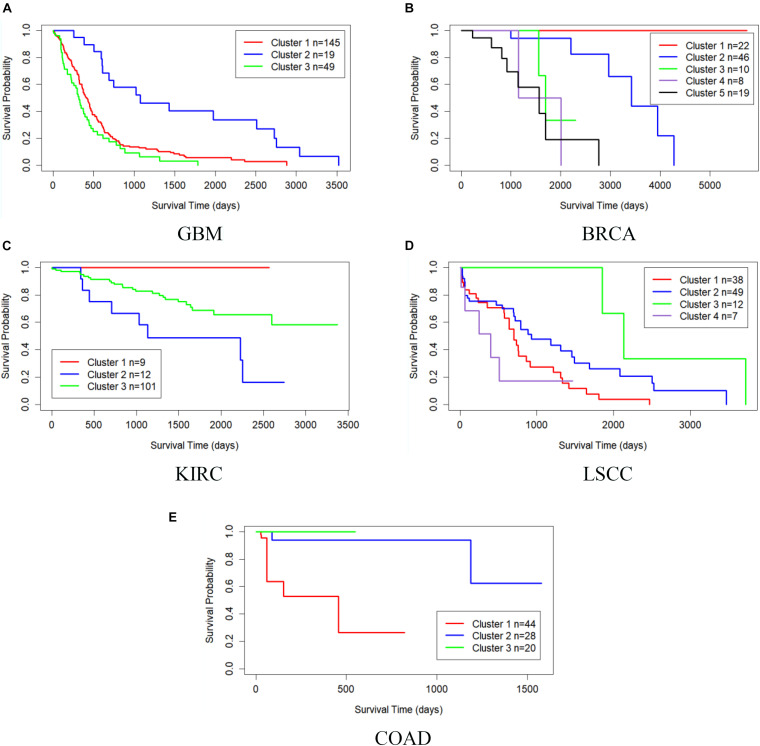
Kaplan–Meier survival curves of MRF-MSC on the cancer types in Wang et al. (2014). **(A)** GBM, **(B)** BRCA, **(C)** KIRC, **(D)** LSCC, and **(E)** COAD.

Table 3 shows the comparison of *P*-values of survival analysis between MRF-MSC and other algorithms on five cancer multi-omics data sets in Wang et al. (2014), respectively. Since SNF is currently recognized as the most representative cancer subtype prediction algorithm, we used the number of clusters suggested in SNF, that is, GBM is clustered into three categories, BRCA is clustered into five categories, KIRC is clustered into three categories, LSCC is clustered into four categories, and COAD clustered into three categories. Compared with other algorithms, MRF-MSC has the lowest *P*-value on all five types of cancer. Figure 2 is the Kaplan–Meier survival analysis curve of MRF-MSC on different cancers. Each curve describes the survival time trend of each cancer subtype. The number of samples in each group is also marked in the figure. Figure 2 shows that MRF-MSC can get significantly different cancer subtypes on all types of cancer.

**TABLE 3:** Comparison of *P*-values of survival analysis between MRF-MSC and other algorithms on five cancer multi-omics data sets in Wang et al. (2014).

Cancer types	Methods					
	MRF-MSC	iClusterPlus	PFA	SNF	ANF	MVSCO
GBM	**1.71E-5**	2.98E-2	1.82E-4	5.01E-5	5.83E-4	1.42E-3
BRCA	**1.31E-5**	5.52E-2	3.10E-4	6.91E-4	3.62E-4	3.54E-4
KIRC	**1.70E-2**	1.14E-1	7.45E-2	2.90E-2	4.97E-2	1.96E-2
LSCC	**6.58E-4**	5.17E-2	1.13E-2	1.10E-2	8.92E-3	9.13E-3
COAD	**8.24E-4**	4.96E-2	6.71E-2	2.42E-3	9.02E-3	8.51E-3

*The best results have been highlighted in bold.*

**FIGURE 3:**
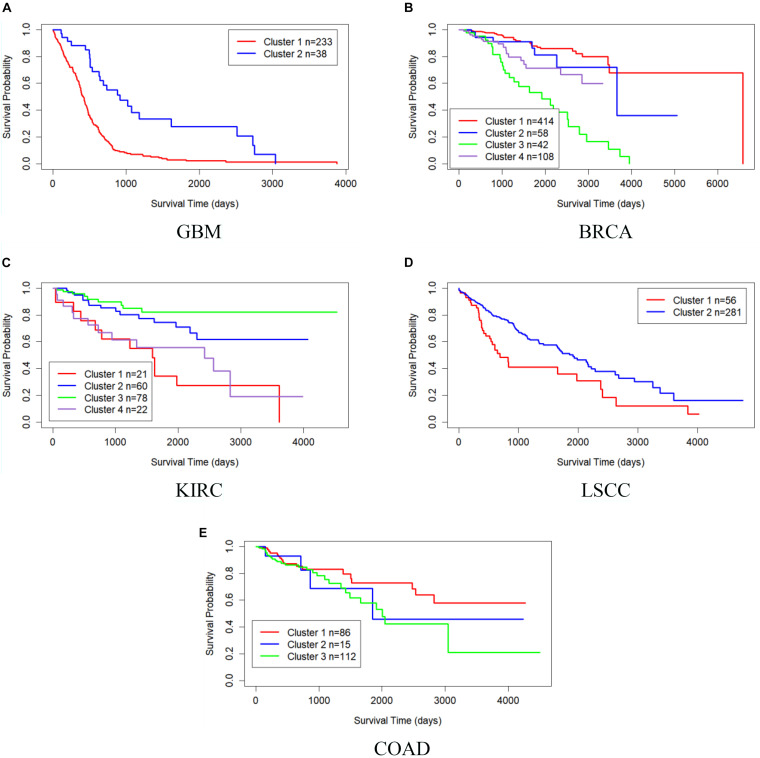
Kaplan–Meier survival curves of MRF-MSC on the cancer types in Rappoport and Shamir (2018). **(A)** GBM, **(B)** BRCA, **(C)** KIRC, **(D)** LSCC, and **(E)** COAD.

Table 4 shows the comparison of *P*-values of survival analysis between MRF-MSC and other algorithms on five cancer multi-omics data sets in Rappoport and Shamir (2018), respectively. These cancer data do not have the number of cancer subtypes available for reference. Therefore, we have to determine the number *k* of these cancer subtypes. iClusterPlus, SNF, and ANF algorithms all have their own way of determining the number of cancer subtypes. For the MRF-MSC, PFA, and MVSCO algorithms, we use Silhouette score (Nguyen et al., 2017) as a reference index for screening the number of cancer subtypes. In the clustering problem, Silhouette analysis is used to study the distance between clusters. Silhouette score measures the closeness of points in the same class compared with points in different classes, which provides a way to evaluate the number of classes. In Table 4, the best *P*-value and the corresponding number of clusters *k* of each algorithm for each cancer type are given. On GBM, BRCA, KIRC, and LSCC data, MRF-MSC algorithm has better experimental results than other algorithms. Figure 3 is the Kaplan–Meier survival analysis curve of MRF-MSC on different cancers. We can find that MRF-MSC can get significantly different cancer subtypes on all types of cancer. All these results demonstrate the effectiveness of the proposed method in cancer subtype prediction.

**TABLE 4:** Comparison of *P*-values of survival analysis between MRF-MSC and other algorithms on five cancer multi-omics data sets in Rappoport and Shamir (2018).

Cancer type	Methods					
	MRF-MSC	iClusterPlus	PFA	SNF	ANF	MVSCO
GBM	**1.43E-6**	3.83E-3	–	7.69E-6	2.17E-1	6.59E-4
	**(*k* = 2)**	(*k* = 10)		(*k* = 2)	(*k* = 3)	(*k* = 2)
BRCA	**5.25E-13**	1.55E-2	3.54E-9	4.38E-9	2.30E-11	4.26E-12
	**(*k* = 4)**	(*k* = 4)	(*k* = 3)	(*k* = 3)	(*k* = 5)	(*k* = 3)
KIRC	**7.10E-6**	2.10E-2	2.93E-3	2.53E-2	4.22E-3	2.71E-4
	**(*k* = 4)**	(*k* = 4)	(*k* = 3)	(*k* = 2)	(*k* = 2)	(*k* = 3)
LSCC	**9.13E-4**	4.63E-3	1.10E-1	9.45E-2	2.19E-2	1.37E-2
	**(*k* = 2)**	(*k* = 3)	(*k* = 3)	(*k* = 2)	(*k* = 2)	(*k* = 2)
COAD	2.63E-1	7.05E-1	3.21E-1	1.52E-1	**7.68E-2**	1.29E-1
	(*k* = 3)	(*k* = 2)	(*k* = 2)	(*k* = 3)	**(*k* = 3)**	(*k* = 2)

*The best results have been highlighted in bold. –Denotes that the algorithm cannot get the clustering result on the data.*

### Analysis on BRCA Data

Breast Invasive Carcinoma refers to a malignant tumor in which cancer cells have penetrated the basement membrane of breast ducts or lobular alveoli and invaded the interstitium. Many scholars have carried out a series of studies and analyses on the gene level, and have given specific subtypes and treatment programs. Based on the microarray predictive analysis model, Parker et al. (2009) proposed a 50-gene classifier (known as PAM50) to classify BRCA into five subtypes: Basal-like, Luminal A, Luminal B, HER2-enriched, and Normal-like. And each subtype is associated with specific mutant genes. For example, there are a large number of PIK3CA mutations in Luminal A and Luminal B, while Basal-like and HER2-enriched are mainly associated with TP53 mutation and ERBB2 amplification, respectively (Koboldt et al., 2012).

**FIGURE 4:**
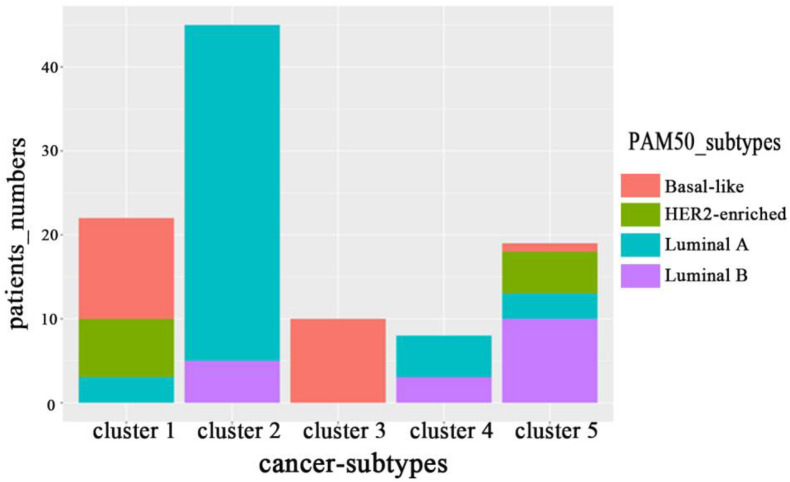
The distribution of subtypes obtained by MRF-MSC on the subtypes: Basal-like, Luminal A, Luminal B and HER2-enriched.

On BRCA data set in Wang et al. (2014), we counted the distribution of clustering results obtained by MRF-MSC on the cancer subtypes: Basal-like, Luminal A, Luminal B, and HER2-enriched in Figure 4. Note that, the clinical information for Normal-like cannot be found in Parker et al. (2009). It can be seen from Figure 4 that Basal-like is mainly distributed in Cluster 1 and Cluster 3, Luminal A is mainly distributed in Cluster 2 and Cluster 4, Luminal B is mainly distributed in Cluster 5, HER2-Enriched is distributed in Cluster 1 and Cluster 5. This shows that the cancer subtypes obtained by MRF-MSC are related to these known cancer subtypes. Furthermore, we counted the distribution of clustering results of MRF-MSC on three susceptible genes: TP53, PIK3CA, and ERBB2 in Table 5. From Table 5 we can find that there are a large number of TP53 mutations which is in line with the characteristics of the Basal-like subtype. The mutation frequency of PIK3CA in Cluster 2 is much higher than the other clusters show that Cluster 2 is related to the known subtypes: Luminal A and Luminal B. The mutations of ERBB2 are mainly distributed on Cluster 1 and Cluster 5, indicating that HER2-enriched subtype is related to Cluster 1 and Cluster 5. The results in Figure 4 and Table 5 are mutually corroborated, proving that MRF-MSC can mine meaningful cancer subtypes.

**TABLE 5:** The distribution of clustering results of MRF-MSC on three susceptible genes: TP53, PIK3CA, and ERBB2.

Susceptible genes	Subtypes predicted by MRF-MSC				
	Cluster 1 (22)	Cluster 2 (46)	Cluster 3 (10)	Cluster 4 (8)	Cluster 5 (19)
TP53	17	8	9	0	5
PIK3CA	8	23	1	1	4
ERBB2	7	3	0	0	7

*The values in this table represent the number of patients counted.*

**FIGURE 5:**
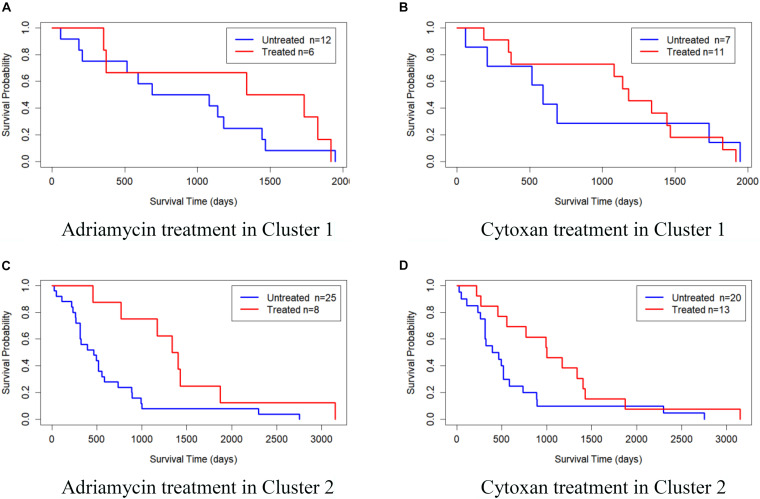
Kaplan–Meier survival curves of drug response in Cluster 1 and Cluster 2. **(A)** Adriamycin treatment in Cluster 1, **(B)** Cytoxan treatment in Cluster 1, **(C)** Adriamycin treatment in Cluster 2, and **(D)** Cytoxan treatment in Cluster 2.

We also validated the obtained subtypes by comparing the survival of different therapeutic agents in each subtype. We downloaded BRCA drug data from TCGA database and selected Adriamycin and Cytoxan for analysis. Since there are few or no samples in Clusters 3, 4, and 5 for these two drugs, we only established a Cox log-rank model on Cluster 1 and Cluster 2 to analyze the quality of drug response. Figure 5 shows the Kaplan–Meier survival curves of drug response in Cluster 1 and Cluster 2. The treated samples and untreated samples are divided into two groups. Clusters 1 and Cluster 2 both responded favorably to Adriamycin and Cytoxan treatment. And the survival of patients with treatment is better than that of patients without treatment. The drug response of Cluster 2 to Adriamycin and Cytoxan (the survival analysis *P*-values of the Cox log-rank model are 9.91 × 10^–3^ and 4.42 × 10^–4^, respectively) is better than that of Cluster 1 (the survival analysis *P*-values of the Cox log-rank model are 0.353 and 0.982, respectively).

**FIGURE 6:**
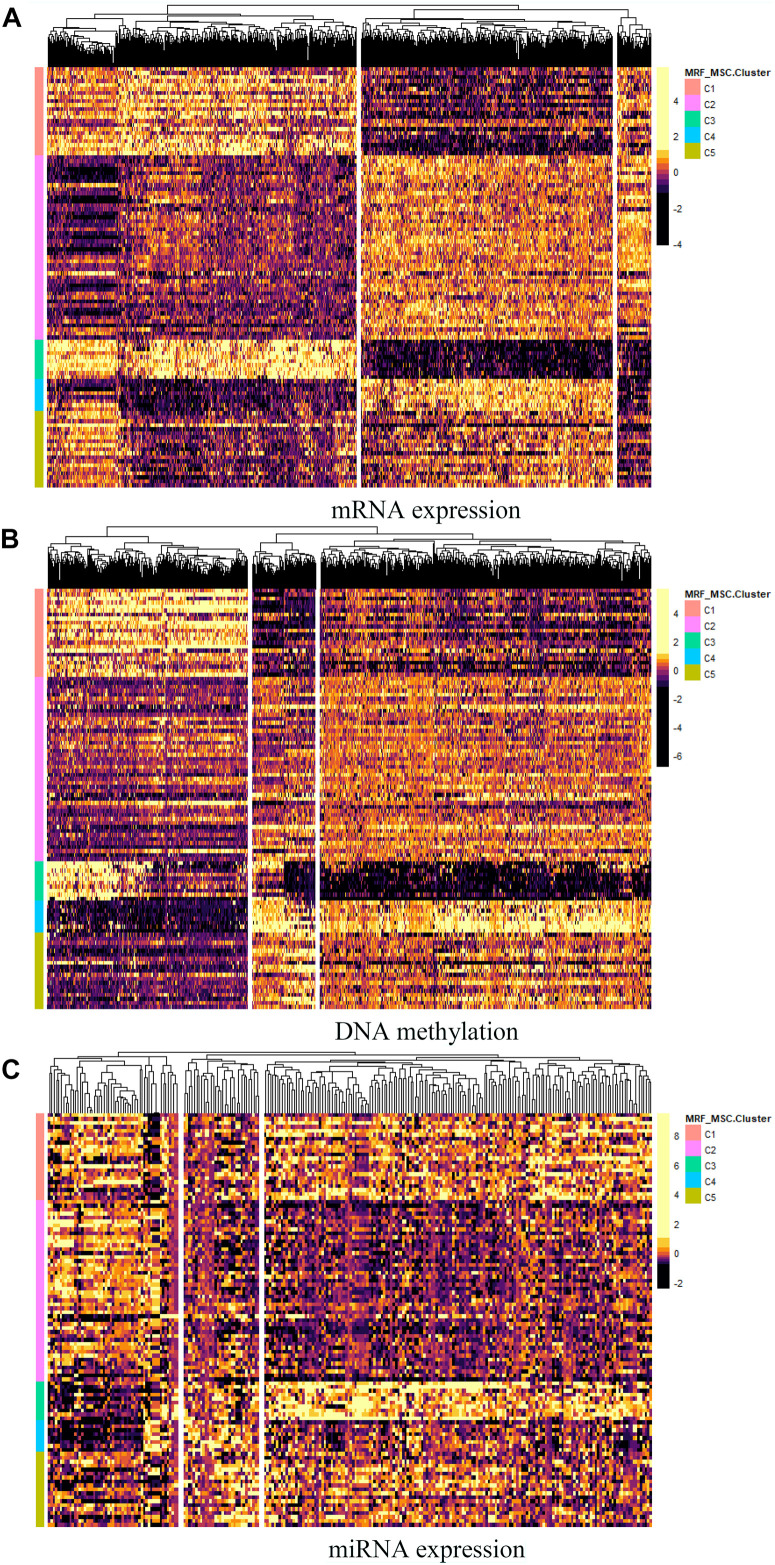
The heatmap of differentially expressed genes in **(A)** mRNA expression, **(B)** DNA methylation, and **(C)** miRNA expression data.

Furthermore, differential expressed genes and GO enrichment analysis on BRCA data are performed to compare differences in characteristics between the five clusters obtained by MSR-MSC. For each omics data, we first used Analysis of Variance (ANOVA) method to select the significant differentially expressed genes in five clusters. And the heatmap of differentially expressed genes in mRNA expression, DNA methylation, and miRNA expression data are shown in Figures 6A–C, respectively. The specific information of these differentially expressed genes can be found in Supplementary File 1. These differentially expressed genes may be closely related to BRCA. For example, the increased expression of GFRA3 (*P*-value = 3.71 × 10^–23^) is associated with lymph node metastasis and advanced tumor stage in BRCA (Wu et al., 2013). mir-186 (*P*-value = 7.41 × 10^–17^) can regulate the migration and erosion of BRCA by PTTG1 (Li et al., 2013), and mir-197 (*P*-value = 2.71 × 10^–17^) targets the tumor-suppressor FUS1 (Du et al., 2009).

**FIGURE 7:**
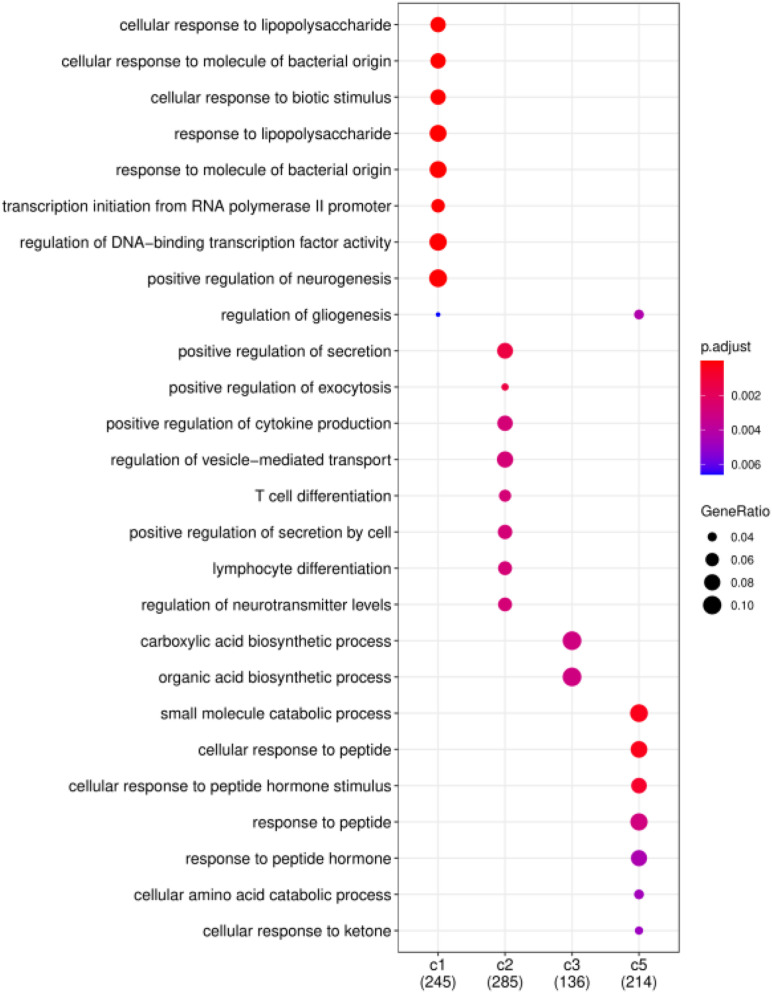
Functional enrichment analysis on BRCA. Significantly different GO biological processes derived from driver genes of different cancer subtypes.

Finally, we consider that the driver genes that affect these five clusters should be different. Therefore, based on the DriverNet method (Bashashati et al., 2012), we use BRCA mutation data, copy number data and mRNA expression data to find the driver genes of each cluster. We screened out the unique driver genes of each cluster to construct GO enrichment analysis (Yu et al., 2012). Figure 7 shows the functional enrichment analysis of four clusters on BRCA. There are too few driver genes in Cluster 4 to form a functional enrichment term. It can be seen that significantly different GO biological processes derived from driver genes of different cancer subtypes (FDR < 0.05). Driver genes in Cluster 1, 2, 3, and 5 are correlated with “cellular response,” “positive regulation,” “biosynthetic process,” and “response to peptide” in GO biological processes, respectively.

## Conclusion

In the past few decades, many multi-view biological data integration models based on graph learning, matrix decomposition, network fusion, deep learning, nuclear methods and other technologies have been designed and applied to a wide range of bioinformatics topics (Li et al., 2016), such as prediction of drug–target interactions (Liu et al., 2021), identification of cancer driver genes (Bashashati et al., 2012) and genotype-phenotype interactions (Qin et al., 2020). These studies provide meaningful insights into the cause and development of cancer. However, how to effectively mine cancer subtypes with biological characteristics from multi-omics data is still a challenging task for bioinformatics. In this paper, a new cancer subtype prediction method was proposed, named as Multi-View Spectral Clustering Based on Multi-smooth Representation Fusion (MRF-MSC). In order to enable the data samples to retain the original feature space and enhance the grouping effect during data representation, we construct smooth representation for each type of data. Then, based on the method of graph fusion, these smooth representations are integrated into one space, and each smooth representation is given a self-weighted weight to measure their contribution. A fused similarity graph with a consistent structure is obtained through optimization. Finally, constrained Laplacian rank is performed on the fused similarity graph, and the label of the sample is obtained through spectral clustering optimization. We use real cancer data sets to demonstrate the capabilities of MRF-MSC. MRF-MSC can effectively integrate the information of multi-omics data, and is superior to several state-of-the-art integration methods in given evaluation indexes. On BRCA data, through various studies, we have verified that the cancer subtypes predicted by MRF-MSC are significantly different and have biological significance.

In addition, we also admit that MRF-MSC has its shortcomings and limitations. It takes a lot of time to select suitable hyperparameters in the optimization process. Moreover, it is not suitable for binary data (somatic mutation), categorical data (copy number states: loss/normal/gain), and it has no ability to find important genes that affect each subtype. Therefore, we will continue to work hard to improve and expand the capabilities of the MRF-MSC algorithm and explore the heterogeneity of cancer.

## Data Availability Statement

The original contributions presented in the study are included in the article/Supplementary Material, further inquiries can be directed to the corresponding author.

## Author Contributions

JL and XW constructed the original idea and designed the experiments. JL and SG wrote the manuscript. JL and YC proofread the manuscript. All authors contributed to the article and approved the submitted version.

## Conflict of Interest

The authors declare that the research was conducted in the absence of any commercial or financial relationships that could be construed as a potential conflict of interest.

## Publisher’s Note

All claims expressed in this article are solely those of the authors and do not necessarily represent those of their affiliated organizations, or those of the publisher, the editors and the reviewers. Any product that may be evaluated in this article, or claim that may be made by its manufacturer, is not guaranteed or endorsed by the publisher.

## Footnotes

1
http://www.cbioportal.org/

## References

[B1] Akbani R. Ng P. K. S. Werner H. M. J. Shahmoradgoli M. Zhang F. Ju Z. (2014). A pan-cancer proteomic perspective on the Cancer genome atlas. *Nat. Commun.* 5 3887–3887. 10.1038/ncomms4887 24871328 PMC4109726

[B2] Bashashati A. Haffari G. Ding J. Ha G. Lui K. Rosner J. (2012). DriverNet: uncovering the impact of somatic driver mutations on transcriptional networks in cancer. *Genome Biol.* 13 1–14. 10.1186/gb-2012-13-12-r124 23383675 PMC4056374

[B3] Bedard P. L. Hansen A. R. Ratain M. J. Siu L. L. (2013). Tumour heterogeneity in the clinic. *Nature* 501 355–364. 10.1038/nature12627 24048068 PMC5224525

[B4] Burrell R. A. McGranahan N. Bartek J. Swanton C. (2013). The causes and consequences of genetic heterogeneity in cancer evolution. *Nature* 501 338–345. 10.1038/nature12625 24048066

[B5] Ding C. H. Q. He X. (2004). “Cluster structure of K-means clustering via principal component analysis,” in *Proceedings of the Pacific-Asia Conference on Advances in Knowledge Discovery and Data Mining*, (Piscataway, NJ: IEEE) 414–418. 10.1007/978-3-540-24775-3_50

[B6] Du L. Schageman J. J. Subauste M. C. Saber B. Hammond S. M. Prudkin L. (2009). miR-93, miR-98, and miR-197 regulate expression of tumor suppressor gene FUS1. *Mol. Cancer Res.* 7 1234–1243. 10.1158/1541-7786.MCR-08-0507 19671678 PMC2741087

[B7] Fan K. (1949). On a theorem of weyl concerning eigenvalues of linear transformations: II^∗^. *Proc. Natl. Acad. Sci. U S A.* 36 31–35. 10.1073/pnas.36.1.31 16588943 PMC1063126

[B8] Feng J. Jiang L. Li S. Tang J. Wen L. (2021). Multi-omics data fusion via a joint kernel learning model for cancer subtype discovery and essential gene identification. *Front. Genet.* 12:647141. 10.3389/fgene.2021.647141 33747053 PMC7969795

[B9] Ge S. Wang X. Cheng Y. Liu J. (2021). Cancer subtype recognition based on laplacian rank constrained multiview clustering. *Genes* 12:526. 10.3390/genes12040526 33916856 PMC8065670

[B10] Goel M. K. Khanna P. Kishore J. (2010). Understanding survival analysis: kaplan-Meier estimate. *Int. J. Ayurveda Res.* 1 274–278. 10.4103/0974-7788.76794 21455458 PMC3059453

[B11] Guo Y. Li H. Cai M. Li L. (2019). Integrative subspace clustering by common and specific decomposition for applications on cancer subtype identification. *BMC Med. Genomics* 12:191. 10.1186/s12920-019-0633-1 31874642 PMC6929329

[B12] Hu H. Lin Z. Feng J. Zhou J. (2014). “Smooth representation clustering,” in *Proceedings of the 2014 IEEE Conference on Computer Vision and Pattern Recognition*, (Piscataway, NJ: IEEE), 3834–3841. 10.1109/CVPR.2014.484

[B13] Kang Z. Shi G. Huang S. Chen W. Pu X. Zhou J. T. (2020). Multi-graph fusion for multi-view spectral clustering. *Knowledge Based Systems* 189 105102. 10.1016/j.knosys.2019.105102

[B14] Koboldt D. C. Fulton R. S. McLellan M. D. Schmidt H. Kalicki-Veizer J. McMichael J. F. (2012). Comprehensive molecular portraits of human breast tumours. *Nature* 490 61–70. 10.1038/nature11412 23000897 PMC3465532

[B15] Li H. Yin C. Zhang B. Sun Y. Shi L. Liu N. (2013). PTTG1 promotes migration and invasion of human non-small cell lung cancer cells and is modulated by miR-186. *Carcinogenesis* 34 2145–2155. 10.1093/carcin/bgt158 23671127

[B16] Li Y. Wu F.-X. Ngom A. (2016). A review on machine learning principles for multi-view biological data integration. *Brief. Bioinform.* 19 325–340. 10.1093/bib/bbw113 28011753

[B17] Liu Z. Chen Q. Lan W. Pan H. Hao X. Pan S. (2021). GADTI: graph autoencoder approach for DTI prediction from heterogeneous network. *Front. Genet.* 12:650821. 10.3389/fgene.2021.650821 33912218 PMC8072283

[B18] Ma T. Zhang A. (2017). “Integrate multi-omic data using affinity network fusion (ANF) for cancer patient clustering,” in *Proceedings of 2017 IEEE International Conference on Bioinformatics and Biomedicine (BIBM)*, (Piscataway, NJ: IEEE), 398–403. 10.1109/BIBM.2017.8217682

[B19] Mantel N. (1966). Evaluation of survival data and two new rank order statistics arising in its consideration. *Cancer Chemotherapy Rep.* 50 163–170. 5910392

[B20] Meng C. Helm D. Frejno M. Kuster B. (2016). MoCluster: identifying joint patterns across multiple omics data sets. *J. Proteome Res.* 15 755–765. 10.1021/acs.jproteome.5b00824 26653205

[B21] Mo Q. Wang S. Seshan V. E. Olshen A. B. Schultz N. Sander C. (2013). Pattern discovery and cancer gene identification in integrated cancer genomic data. *Proc. Natl. Acad. Sci. U S A.* 110 4245–4250. 10.1073/pnas.1208949110 23431203 PMC3600490

[B22] Ng A. Y. Jordan M. I. Weiss Y. (2001). On spectral clustering: analysis and an algorithm. *Neural Inform. Process. Systems* 14 849–856.

[B23] Nguyen T. Tagett R. Diaz D. Draghici S. (2017). A novel approach for data integration and disease subtyping. *Genome Res.* 27 2025–2039. 10.1101/gr.215129.116 29066617 PMC5741060

[B24] Nie F. Li J. Li X. (2016). “Parameter-free auto-weighted multiple graph learning: a framework for multiview clustering and semi-supervised classification,” in *Proceedings of the 25th International Joint Conference on Artificial Intelligence*, (Piscataway, NJ: IEEE), 1881– 1887.

[B25] Nie F. Li J. Li X. (2017). “Self-weighted multiview clustering with multiple graphs,” in *Proceedings of 26th International Joint Conference on Artificial Intelligence*, (Piscataway, NJ: IEEE), 2564–2570. 10.24963/ijcai.2017/357

[B26] Parker J. S. Mullins M. Cheang M. C. U. Leung S. Voduc D. Vickery T. (2009). Supervised risk predictor of breast cancer based on intrinsic subtypes. *J. Clin. Oncol.* 27 1160–1167. 10.1200/JCO.2008.18.1370 19204204 PMC2667820

[B27] Qin Z. Su J. Li M. Yang Q. Yi S. Zheng H. (2020). Clinical and genetic analysis of CHD7 expands the genotype and phenotype of charge syndrome. *Front. Genet.* 11:592. 10.3389/fgene.2020.00592 32625235 PMC7314916

[B28] Rappoport N. Shamir R. (2018). Multi-omic and multi-view clustering algorithms: review and cancer benchmark. *Nucleic Acids Res.* 46 10546–10562. 10.1093/nar/gky889 30295871 PMC6237755

[B29] Schuster S. C. (2008). Next-generation sequencing transforms today’s biology. *Nat. Methods* 5 16–18. 10.1038/nmeth1156 18165802

[B30] Shen R. Olshen A. B. Ladanyi M. (2010). Integrative clustering of multiple genomic data types using a joint latent variable model with application to breast and lung cancer subtype analysis. *Bioinformatics* 26 292–293. 10.1093/bioinformatics/btp659 PMC2800366 19759197

[B31] Shi Q. Zhang C. Peng M. Yu X. Zeng T. Liu J. (2017). Pattern fusion analysis by adaptive alignment of multiple heterogeneous omics data. *Bioinformatics* 33 2706–2714. 10.1093/bioinformatics/btx176 28520848

[B32] Wang B. Mezlini A. M. Demir F. Fiume M. Tu Z. Brudno M. (2014). Similarity network fusion for aggregating data types on a genomic scale. *Nat. Methods* 11 333–337. 10.1038/nmeth.2810 24464287

[B33] Wu Z. Pandey V. Wu W. Y. Ye S. Zhu T. Lobie P. E. (2013). Prognostic significance of the expression of GFRalpha1. GFRalpha3 and Syndecan. *BMC Cancer* 13:34. 10.1186/1471-2407-13-34 23351331 PMC3562211

[B34] Yu G. Wang L. G. Han Y. He Q. Y. (2012). clusterProfiler: an R package for comparing biological themes among gene clusters. *Omics J. Int. Biol.* 16 284–287. 10.1089/omi.2011.0118 22455463 PMC3339379

[B35] Yu Y. Zhang L.-H. Zhang S. (2019). Simultaneous clustering of multiview biomedical data using manifold optimization. *Bioinformatics* 35 4029–4037. 10.1093/bioinformatics/btz217 30918942

[B36] Zhang S. Liu C.-C. Li W. Shen H. Laird P. W. Zhou X. J. (2012). Discovery of multi-dimensional modules by integrative analysis of cancer genomic data. *Nucleic Acids Res.* 40 9379–9391. 10.1093/nar/gks725 22879375 PMC3479191

